# Brugada syndrome in a patient with amyotrophic lateral sclerosis: a case report

**DOI:** 10.1186/s13256-017-1356-6

**Published:** 2017-07-14

**Authors:** Anusha Battineni, Rohit Gummi, Naresh Mullaguri, Raghav Govindarajan

**Affiliations:** Department of Neurology, University of Missouri Healthcare, 5 Hospital Drive, CE 540, Columbia, MO 65201 USA

**Keywords:** Amyotrophic lateral sclerosis, Brugada syndrome, Cardiac arrest

## Abstract

**Background:**

Amyotrophic lateral sclerosis is a fatal neuromuscular disorder characterized by progressive death of the upper and lower motor neurons in the central nervous system. Patients with this disease die mostly as a result of respiratory failure; however, owing to prolonged survival through assisted ventilation, cardiovascular causes are increasingly responsible for mortality. We report what is to the best of our knowledge the first case of type 2 Brugada syndrome causing ventricular tachyarrhythmia and cardiac arrest in a patient with upper limb onset amyotrophic lateral sclerosis.

**Case presentation:**

A 48-year-old Caucasian woman with a significant past medical history of papillary thyroid carcinoma status postresection, pulmonary embolism on anticoagulation, and a recent diagnosis of right upper limb-onset amyotrophic lateral sclerosis presented to the emergency department of our hospital with acute on chronic shortness of breath. On further evaluation, she was found to have hypoxic and hypercapnic respiratory failure and was placed on bilevel positive airway pressure ventilation. Her 12-lead electrocardiogram showed sinus rhythm with J-point elevation, saddle-shaped ST segment elevation, predominantly in V1 and V2 with no significant QTc prolongation. No troponin elevation was noted in her laboratory workup. Because she was unable to protect her airway, a decision was made to intubate her. After 1 minute of induction with etomidate and succinylcholine, she went into pulseless ventricular tachycardia and fibrillation requiring three cycles of cardiopulmonary resuscitation with high-quality chest compressions, three doses of epinephrine, and a loading dose of amiodarone prior to return of spontaneous circulation. She was further evaluated by cardiology services and was diagnosed with type 2 Brugada syndrome, for which she was started on quinidine. Her respiratory failure and the drugs she received for intubation likely caused her ventricular tachycardia to occur in conjunction with an underlying Brugada pattern seen on an electrocardiogram. The results of evaluation of her genetic panel for Brugada syndrome were negative. She was subsequently discharged to home in stable condition after a 10-day hospital stay.

**Conclusions:**

Amyotrophic lateral sclerosis is a progressive neuromuscular disorder with significant mortality. Respiratory failure is the leading cause of death, but lately, owing to increased survival associated with early tracheostomy and positive pressure ventilation, there has been an increasing trend in the identification of cardiovascular causes of mortality, especially arrhythmias, that may need periodic electrocardiographic surveillance.

## Background

Amyotrophic lateral sclerosis (ALS) is a fatal neuromuscular disorder. It is characterized by progressive death of the upper and lower motor neurons in the central nervous system, resulting in weakness and atrophy of the bulbar muscles and muscles of the upper and lower extremities. Death in patients with ALS is caused primarily by respiratory muscle weakness, but there has been a recent surge in cardiovascular causes of mortality as these patients survive longer with the help of mechanical ventilation. Sudden cardiac death has been reported in patients with different types of motor neuron diseases, such as ALS and Kennedy’s disease, suggesting their multiorgan involvement. Although Kennedy’s disease is known to be associated with different types of Brugada syndrome, there has been no reported case of its association in a patient diagnosed with ALS. To the best of our knowledge, this is the first case report of a patient with upper limb-onset ALS with ventricular tachyarrhythmia secondary to type 2 Brugada syndrome.

## Case presentation

### Patient information

A 48-year-old Caucasian woman presented to a neurology clinic for progressive right upper limb weakness that had started 3 months earlier. Her major complaints were frequently dropping things from her hands, worsening handwriting, difficulty holding a spoon, and inability to do her job as a desk clerk. She also noticed some twitching in the muscles of her right upper and lower extremities. She denied numbness or tingling in the extremities and weakness of the bulbar muscles (dysphagia, dysarthria, and shortness of breath). She denied any neck pain; trauma; and constitutional symptoms such as fever, night sweats, and weight loss. Her past medical history included rosacea and dry eyes. She had undergone a cesarean section and plantar fasciotomy in the past. Her father had died as a result of heart failure. She reported no neurodegenerative diseases, neuromuscular diseases, or sudden deaths in other family members. She had no history of smoking or alcohol or illicit drug abuse and no known drug allergies.

### Physical examination

On examination, she did not have any cognitive dysfunction, and the result of her cranial nerve examination was normal. Her motor examination revealed decreased strength in both proximal and distal muscle groups, predominantly in the intrinsic hand muscles with wasting and in the shoulder abductors. Fasciculations were noted in the right upper and lower extremities in various muscle groups. Her deep tendon reflexes were brisk with positive jaw jerk, Hoffman’s sign on the left, and bilateral ankle clonus. Her plantar reflexes were mute bilaterally. Her sensory system, coordination, and gait were unremarkable. Magnetic resonance imaging of the brain and the cervical and thoracic spine was unremarkable. The result of her autoimmune and paraneoplastic workup was negative, although the result of her malignancy workup was positive for papillary thyroid cancer, which was subsequently resected. An electromyogram with nerve conduction studies showed denervation in three body regions, consistent with the diagnosis of ALS. During the next 3 months, her pulmonary function tests showed a decrement by more than 20% in measurements of predicted forced vital capacity and forced expiratory volume in 1 second. One month later, she had an acute episode of shortness of breath and was diagnosed with pulmonary embolism, for which she was started on anticoagulation.

**Fig. 1:**
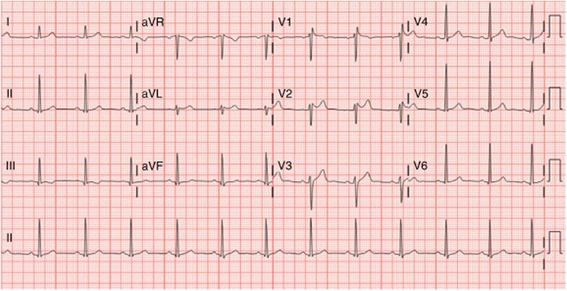
Twelve-lead electrocardiogram showing type 2 Brugada pattern. Saddleback appearance can be seen in the precordial leads, especially in lead V2. This is characterized by a ≥2-mm ST elevation followed by a trough ≥1 mm and either a positive or biphasic T wave

Nine months into her diagnosis of ALS, she presented to the emergency department of our hospital with acute on chronic shortness of breath and was hypoxic and hypercapnic with a negative inspiratory pressure of −10 mmHg, requiring bilevel positive airway pressure ventilation (BiPAP). In further evaluation for cardiopulmonary causes, her chest x-ray was unremarkable; a computed tomographic angiogram of the chest ruled out pulmonary embolism; and an electrocardiogram showed sinus rhythm with J-point elevation, saddle-shaped ST segment elevation predominantly in V1 and V2 with no significant QTc prolongation and negative troponinemia (Fig. 1). Eventually, she became lethargic with a worsening Glasgow Coma Scale score and increased work of breathing, and she was unable to protect her airway, requiring intubation. She was continued on BiPAP until induction for intubation. Etomidate and succinylcholine were administered, and within 1 minute into induction, she developed pulseless ventricular tachycardia and ventricular fibrillation. Advanced cardiac life support was initiated with defibrillation, three rounds of epinephrine, and a loading dose of amiodarone prior to return of spontaneous circulation. After some time, she became responsive and started following simple commands, and a decision was made not to use a hypothermia protocol. She was admitted to the cardiac intensive care unit for close monitoring. Her echocardiogram showed that her left ventricular ejection fraction was 70%, and asymmetric hypertrophy of basal segments of the left ventricle was noted. She was diagnosed with type 2 Brugada syndrome by cardiology services as the cause of her ventricular tachyarrhythmia, and she was started on quinidine. The results of her genetic panel for Brugada syndrome was negative. There were no recurrences of ventricular arrhythmias during the rest of her hospitalization. She declined automatic implantable cardioverter-defibrillator placement and was later discharged to home hospice care with a tracheostomy.

## Discussion

ALS is currently considered a multisystem disorder on the basis of its involvement of other organ systems leading to significant mortality and morbidity [1–5]. Most patients with this disease experience respiratory failure secondary to progressive chest wall weakness and pulmonary embolism resulting from their immobility. In our patient’s case, we emphasize that cardiovascular involvement may also contribute to the disease mortality. In a French ALS study by Gil *et al*., who researched the leading causes of mortality, 3.4% of patients died as a result of myocardial infarction or dysrhythmias, and a few patients had sudden cardiac death [3]. The cardiovascular aberrations, such as QT prolongation, heart rate-blood pressure dyssynchrony, and nocturnal hypotension during the course of the disease have frequently been reported in this patient population. This could be due to decreasing sympathetic tone secondary to degeneration of the intermediolateral column in the upper levels of the spinal cord, as previously shown in pathological studies by Hirohide *et al*. [6]. Brugada syndrome is an inherited arrhythmia syndrome characterized by coved (type 1) or saddleback (types 2 and 3) ST-segment elevations (≥2 mm), followed by deep T-wave inversions in leads V1–V3. It is associated with increased risk of sudden cardiac death, especially in young male adults of Southeast Asian origin. Although genetic mutations in several sodium and calcium channel genes have been identified, an imbalance in sympathetic and parasympathetic nervous systems in the pathogenesis of Brugada syndrome has been proposed [7, 8]. Brugada syndrome, which can predispose patients to develop ventricular tachyarrhythmia and circulatory collapse, has not been reported in patients with ALS, which can predispose them to develop ventricular tachyarrhythmia and circulatory collapse, as happened in our patient. Kennedy’s disease, another type of motor neuron disease, is known to be associated with all types of Brugada syndrome [4]. We hypothesize that patients with ALS are also at high risk for this pattern, given the changes in the efferent sympathetic dysfunction shared with Kennedy’s disease. Our patient did not have any preexisting mutations to develop Brugada syndrome, ruling out the possibility of a casual association, but rather had an important pathophysiological mechanism of motor neuron diseases that needs to be explored further. Because patients with ALS tend to live longer with improved respiratory management, identifying and managing potential causes of acute cardiorespiratory failure, such as ventricular tachyarrhythmias, need further emphasis. Periodic surveillance with electrocardiography to identify patterns of Brugada syndrome and QTc prolongation may be considered to identify patients at high risk for potential cardiac arrest.

## Conclusions

Cardiovascular causes, especially malignant arrhythmias, can also be associated with significant mortality in patients with ALS. We propose periodic electrocardiographic surveillance in patients with ALS to identify abnormal patterns such as Brugada syndrome.
